# Age Difference in the Association Between Nutritional Status and Dynapenia in Older Adults

**DOI:** 10.3390/nu17040734

**Published:** 2025-02-19

**Authors:** Chih-Ching Chang, Ting-Fu Lai, Jiaren Chen, Yung Liao, Jong-Hwan Park, Yen-Jung Chang

**Affiliations:** 1 Department of Health Promotion and Health Education, College of Education, National Taiwan Normal University, Taipei 106308, Taiwan; 81305010e@ntnu.edu.tw; 2 Graduate Institute of Sport, Leisure and Hospitality Management, National Taiwan Normal University, Taipei 106308, Taiwan; 80905010e@ntnu.edu.tw (T.-F.L.); 80931005a@ntnu.edu.tw (J.C.); liaoyung@ntnu.edu.tw (Y.L.); 3 Department of Convergence Medicine, Pusan National University School of Medicine, Yangsan 50612, Republic of Korea; 4 Department of Clinical Bio-Convergence, Graduate School of Convergence in Biomedical Science, Pusan National University School of Medicine, Yangsan 50612, Republic of Korea; 5 Convergence Medical Institute of Technology, Pusan National University Hospital, Busan 49241, Republic of Korea

**Keywords:** malnutrition, undernutrition, handgrip strength, physical function, sarcopenia, aging

## Abstract

Background: Although nutritional status plays a critical role in maintaining muscle strength, limited evidence exists regarding its association with dynapenia. Objectives: We aimed to investigate the association between different nutritional statuses and dynapenia among Taiwanese older adults, and assessed whether age modifies this relationship. Methods: In this study, we enrolled individuals aged 65 years and older living in community settings through convenience sampling from 2020 to 2021, following a cross-sectional design. The Mini-Nutritional Assessment Short Form (MNA-SF) was used to assess whether the participants were at nutritional risk. Standardized assessments measured muscle strength (handgrip measurement), physical performance (6 m walking test), and muscle mass (bioelectrical impedance analysis) to confirm dynapenia classifications. The interaction terms were tested using likelihood ratio tests to examine for dynapenia between nutritional status and age. For overall sample and subgroup analyses, binary logistic regression was employed. Results: Among 211 participants (mean age: 80.7 ± 7.1 years), after adjusting for potential confounders, those at nutritional risk (OR: 3.11; 95% CI: 1.31–7.36) were positively associated with dynapenia, whereas higher MNA-SF scores (OR: 0.73; 95% CI: 0.57–0.93) were negatively associated. Interactions regarding dynapenia were observed between nutritional status and age group (*p* = 0.014), with nutritional risk significantly associated with dynapenia only in the old–old group (≥75 years) (OR = 4.11, 95% CI: 1.39–12.15). Conclusions: Age is a potential moderator of nutritional status and dynapenia among older populations. Nutritional status appeared to be more profound in the old–old group in terms of the risk of dynapenia. These findings offer insights for monitoring nutritional status and implementing targeted interventions to prevent dynapenia in those aged over 75 years. Future studies using prospective designs should explore the underlying mechanisms linking nutritional status to dynapenia and assess the effectiveness of nutritional interventions in preventing muscle strength decline.

## 1. Introduction

The rapidly growing aging population is contributing to an increased occurrence of age-related health outcomes [1], leading to the global burden of disability and morbidity among older populations [2]. Research indicates that disability and mortality in older adults shows a stronger association with reduced muscle strength than with muscle mass [3,4,5]. Declining muscle strength not only elevates the likelihood of frailty, but also increases the risk of cardiovascular disease and all-cause mortality [6,7,8], underscoring its critical role in maintaining physical abilities in older adults. Dynapenia is characterized by age-associated reductions in muscle strength [9,10] and could be a preliminary indicator for screening sarcopenia [11]. Furthermore, dynapenia has a stronger association with physical ability limitations than that of sarcopenia [12] and correlates with a higher risk of mortality [3,13]. The prevalence of dynapenia varies worldwide, ranging from 10% to 84.6% [14,15,16,17]. In Taiwan, it is approximately 28.6% to 31.3% [18,19], and a previous 6-year follow-up study reported that nearly 18.5% of community-dwelling older adults transitioned from a healthy state to dynapenia, suggesting that deterioration in muscle function could be more common [20]. Given these findings, a better comprehension of the modifiable factors associated with dynapenia is crucial, not only to facilitate early identification, but also to reduce its impact on physical ability outcomes among older adults.

Nutritional status, a modifiable lifestyle factor, and particularly adequate protein and essential nutrient intake is critical for promoting muscle protein synthesis among older populations [10,21,22]. However, malnutrition is prevalent in this population, largely because of several age-related changes that elevate the risk of nutritional deficits [23,24]. Such nutritional deficits can contribute to decreased physical functionality, poor quality of life, and increased mortality [25,26,27]. Although previous research has established nutritional status as a key factor influencing muscle strength [28,29] and physical performance [23,30], current evidence on the correlation between nutritional status and dynapenia remains limited and is primarily focused on specific populations, such as individuals with Parkinson’s disease [31], post-COVID-19 [32], and postmenopausal women [33]. This focus on specific populations highlights a research gap in understanding this relationship in the community-dwelling older population, a broader at-risk population. Moreover, prior research [20] has indicated that dynapenia has gradually become a health concern in the aging population of Taiwan. Despite this concerning trend, a deficiency of localized research exists exploring the relationship between nutritional status and dynapenia among Taiwanese older adults in community settings. Addressing this gap is essential for developing targeted nutritional interventions aimed at mitigating the impact of dynapenia.

Furthermore, prior research has identified age as a crucial factor of malnutrition among older populations, with the prevalence rates increasing as the age advances due to physiological changes, decreased appetite, and reduced nutrient absorption [34,35,36]. Additionally, muscle strength declines progressively with aging [37], and this decline accelerates beyond the age of 60 at a rate of approximately 3% per year [38]. These age-related physiological changes may further contribute to muscle loss and exacerbate the negative impact of malnutrition on muscle function. Given this age-related risk, we hypothesized that the association between nutritional status and dynapenia differs between the young–old group (comprising older adults aged 65–74 years) and the old–old group (comprising those aged ≥ 75 years) [39,40]. Therefore, the purpose of this study was to explore the relationship between different nutritional statuses (normal/at risk) and dynapenia among Taiwanese older adults living in community settings, as well as to assess whether age serves as a moderator of this relationship.

## 2. Materials and Methods

### 2.1. Research Design and Study Samples

In this study, we employed a cross-sectional design and recruited participants aged over 65 years living in community settings using convenience sampling from the Department of Geriatrics and Gerontology at National Taiwan University Hospital (NTNU). From September 2020 to December 2021, participants were recruited through two methods. One method involved the preliminary screening of potentially eligible individuals who had completed periodic physical examinations, whereas the other method identified eligible individuals attending the outpatient clinic through referrals from outpatient physicians.

The participants first completed a series of questionnaires through in-person interviews to report their sociodemographic information, health behaviors, and health status, with the Mini-Nutritional Assessment Short Form (MNA-SF) utilized to assess whether they were at nutritional risk. Next, standardized procedures were employed to measure handgrip strength, gait speed, and muscle mass to assess whether the participants met the dynapenia criteria outlined by the 2019 Asian Working Group for Sarcopenia (AWGS) guidelines. Finally, to objectively record moderate-to-vigorous physical activity (MVPA), participants were instructed to wear a triaxial accelerometer for at least 10 h/day over 7 consecutive days.

**Figure 1:**
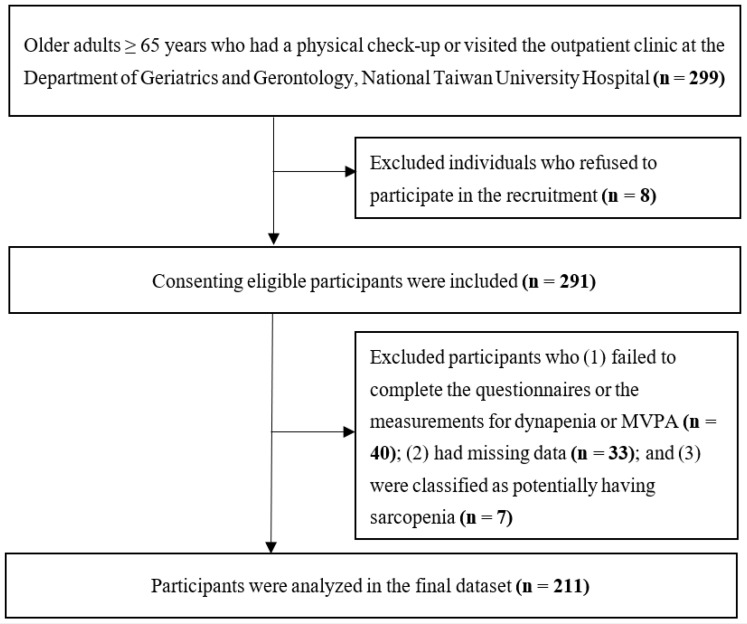
Study process flowchart.

The minimum sample size for a binary logistic regression analysis was estimated to be 131, with an alpha level (α) of 0.05 and a statistical power of 0.8, calculated using G*Power version 3.1.9.7 [41]. Given the possibility of missing data, we initially recruited 299 participants; however, eight individuals declined to participate, resulting in 291 participants being enrolled. Subsequently, the participants were screened according to the following exclusion criteria: (1) inability to complete the questionnaires, failure to follow the standard measurement procedures for dynapenia, or noncompliance with wearing the triaxial accelerometer (n = 40); (2) incomplete or missing data from the questionnaires or MVPA records (n = 33); and (3) being classified as potentially having sarcopenia (n = 7). In the final analysis, 211 participants were included (Figure 1).

The rights of all participants were safeguarded through the acquisition of signed informed consent forms before the study’s initiation. At the completion of the study, each participant was given a US$7 convenience store gift voucher in appreciation. The approval for the study protocol was granted by the Research Ethics Committee of National Taiwan University Hospital (REC number: 202008046RINC).

### 2.2. Measurements

#### 2.2.1. Nutritional Status

This study applied the MNA-SF, a tool developed for older adults aged over 65 years, to evaluate the participants’ nutritional status [42,43,44]. The MNA-SF assesses an individual’s changes over the past three months in degrees of weight loss, food intake, physical activity level, body mass index (BMI), and presence of psychiatric problems or psychological stress [43]. The total MNA-SF score ranges from 0 to 14, where higher scores indicate a lower nutritional risk. The MNA-SF defines scores of >11 as normal and ≤11 as at nutritional risk [43].

#### 2.2.2. Dynapenia

The definition of dynapenia was low muscle function, indicated by reduced muscle strength and/or poor physical performance, while maintaining normal muscle mass [45]. Muscle strength was assessed by handgrip measurements, physical performance by a 6 m walking test, and appendicular skeletal muscle mass (ASM) by bioelectrical impedance analysis (BIA). The cutoffs for these measurements were based on the 2019 updated AWGS guidelines [11]. Therefore, the participants were classified as having dynapenia if they exhibited reduced handgrip strength (<28.0 kg for men; <18.0 kg for women) or a gait speed of <1.0 m/s (for both sexes), while maintaining normal ASM (≥7.0 kg/m^2^ for men; ≥5.7 kg/m^2^ for women). The participants with normal ASM and muscle function were classified as not having dynapenia. Additionally, to allow a clear comparison between the participants with dynapenia and without (healthy individuals), those with decreased ASM (<7.0 kg/m^2^ for men; <5.7 kg/m^2^ for women), who may be classified as potentially having sarcopenia [11], were excluded.

#### 2.2.3. Muscle Strength

To evaluate muscle strength, a hydraulic handheld dynamometer (Jamar Plus+ Digital Hand Dynamometer; Lafayette Instrument Company, Lafayette, IN, USA) was utilized for the handgrip measurements. The participants were asked to perform at maximum strength to grip the device with their dominant hand, and the highest handgrip strength recorded from the two trials was used for the analysis.

#### 2.2.4. Physical Performance

Physical performance was measured as the walking speed of the participants using a 6 m walking test. Each participant completed the walk twice under safe conditions, and the shorter of the two was recorded for the analysis.

#### 2.2.5. Muscle Mass

A bioelectrical impedance analyzer (DC-430MA; TANITA, Tokyo, Japan) was utilized to measure ASM. We instructed the participants to stand barefoot on the analyzer and maintain a firm grip on the device handles throughout the measurement process to ensure accurate results.

#### 2.2.6. Covariates

We collected sociodemographic data including age (categorized as 65 to 74 years or 75 years and above), sex (men or women), educational level (less than university level or university-educated and above), and living status (living alone or with others). The assessment of health behaviors focused on documenting the participants’ smoking and alcohol consumption habits over the past year, with the responses categorized as either “yes” or “no”. The health status parameters, including BMI, were calculated as weight (in kilograms) divided by height (in meters) squared (kg/m^2^). MVPA was objectively measured using a triaxial accelerometer (GT3X+ ActiGraph; Pensacola, FL, USA) worn over 7 consecutive days to accurately assess the average weekly minutes of MVPA [46]. The threshold for MVPA was set based on the World Health Organization (WHO) recommendations, advising that older populations should achieve a minimum of 150 min weekly of MVPA [47].

### 2.3. Statistical Analysis

The basic characteristics of the participants were presented using descriptive statistics. We conducted chi-square tests and t-tests to assess dynapenia in relation to categorical and continuous variables, respectively, and the significant covariates were adjusted in the logistic regression analyses. Binary logistic regression analyses, including both unadjusted and adjusted models, were performed to examine the relationship between different nutritional statuses and dynapenia in the overall sample. Likelihood ratio tests were performed to determine whether interaction terms existed for dynapenia between the nutritional status and the characteristic variables, particularly the age groups. Upon confirming a significant interaction, the sample was stratified into two age groups: old–old (75+ years) and young–old (65–74 years) [39,40]. Finally, binary logistic regression analyses were performed for the two age groups as subgroup analyses to examine the relationship between different nutritional statuses and dynapenia. The results were presented as odds ratios (ORs) along with 95% confidence intervals (CIs). IBM SPSS (version 23.0, Chicago, IL, USA) was applied, and the statistical significance level was established at *p* < 0.05.

## 3. Results

### 3.1. Sample Characteristics

**Figure 2:**
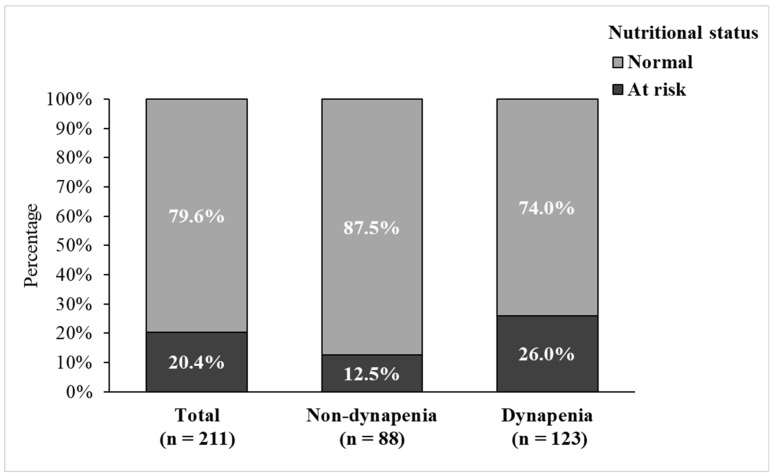
Distribution of nutritional status among total participant, non-dynapenia, and dynapenia groups.

Table 1 summarizes an overview of the participants’ sociodemographic, health behavior, and health status variables. Among the 211 participants, the mean age was 80.7 ± 7.1 years; 46.0% were men, 51.2% had an education level below university, and 90.5% lived with others. Regarding health behaviors, most participants did not use tobacco (91.9%) or alcohol (89.6%). Of the participants, 31.3% engaged in a minimum of 150 min of MVPA weekly. In terms of health status, the mean BMI was 24.4 ± 3.7 kg/m^2^ for all participants. The mean MNA-SF scores were 12.6 ± 1.7, and 43 (20.4%) participants were at nutritional risk. There were 123 (58.3%) participants who were classified as having dynapenia. The results of the chi-square and t-tests showed that nutritional status, educational level, BMI, smoking, and MVPA were significantly associated with dynapenia. Moreover, a greater proportion of the participants at nutritional risk were distributed within the dynapenia group (Figure 2).

### 3.2. Associations Between MNA-SF, Nutritional Status, and Dynapenia Among All Participants

**Figure 3:**
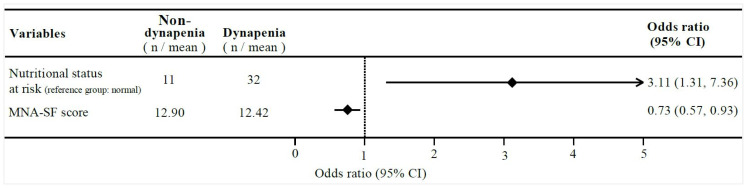
Adjusted odds ratios for association between nutritional status, MNA-SF scores, and dynapenia.

Table 2 shows the associations between nutritional status (normal or at risk), MNA-SF scores, and dynapenia across the two logistic regression models for the entire sample. Model 1 represented a crude model. Model 2 accounted for adjustments related to age, sex, educational level, BMI, smoking status, and MVPA. Both the nutritional status at risk (model 1: OR: 2.46; 95% CI: 1.16–5.21; *p* = 0.018, model 2: OR: 3.11; 95% CI: 1.31–7.36; *p* = 0.009) and the MNA-SF scores (model 1: OR: 0.83; 95% CI: 0.70–1.00; *p* = 0.045, model 2: OR: 0.73; 95% CI: 0.57–0.93; *p* = 0.011) demonstrated a significant association with dynapenia in the unadjusted and adjusted models. Figure 3 illustrates the results from model 2, showing the OR and 95% CI for the associations between the nutritional status at risk, MNA-SF scores, and dynapenia.

### 3.3. Interactions Regarding Dynapenia Between Nutritional Status and Sociodemographic Variants

Table 3 shows the significant interactions regarding dynapenia between nutritional status and age (*p* = 0.014).

### 3.4. Associations Between Nutritional Status and Dynapenia in Different Age Groups

**Figure 4:**
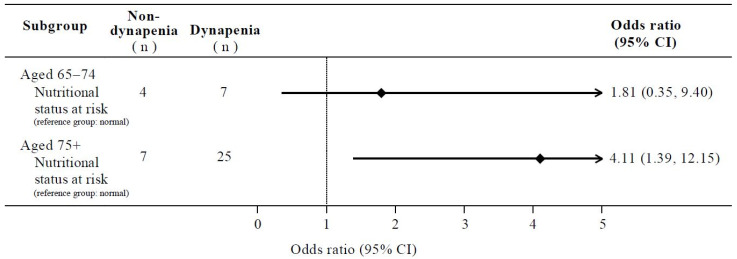
Adjusted odds ratios for association between nutritional status at risk and dynapenia across age groups.

Table 4 shows the associations between the nutritional status at risk and dynapenia in different age groups. Model 1 represented a crude model, whereas Model 2 was an adjusted model (adjusted for sex, educational level, BMI, smoking status, and MVPA). In the aged 65–74 group (n = 53), nutritional status was not significantly associated with dynapenia in both models, whereas in the aged 75+ group (n = 158), the nutritional status at risk was significantly associated with dynapenia, with an OR of 3.15 (95% CI: 1.27–7.80; *p* = 0.013) in model 1 and 4.11 (95% CI: 1.39–12.15; *p* = 0.010) in model 2. Figure 4 illustrates the results from model 2, presenting the OR and 95% CI for the association between the nutritional status at risk and dynapenia, stratified by age groups.

## 4. Discussion

The present study is the first to explore the relationship between different nutritional statuses and dynapenia, based on the 2019 AWGS criteria, among older adults from community settings in Taiwan and to assess whether age is a potential modifier of this relationship. The primary findings indicate that, in the overall sample, being at nutritional risk is correlated with a higher risk of dynapenia. Furthermore, the study provides evidence of age as a potential moderator of these associations, showing a significant correlation in the old–old group, but not in the young–old group. Regarding the goal of dynapenia prevention, our results indicate that public health and nutrition practitioners should develop an age-specific intervention strategy to enhance nutritional status, especially in the old–old population.

Consistent with previous research [31,32,33], our findings reveal an association between nutritional risk and dynapenia. Several studies from different regions of the world have also explored this relationship in distinct populations. While our study examines community-dwelling older adults, a Brazilian study indicated that in patients with Parkinson’s disease, dynapenia is associated with a reduced calf circumference and an increased body fat percentage [31]. In line with this, an Italian study highlighted a high prevalence of malnutrition and dynapenia in post-COVID-19 patients [32]. Moreover, a Canadian study emphasized the nutritional aspect, particularly protein intake, as a key modifiable factor influencing dynapenia among postmenopausal women [33]. Our findings align with these international studies; however, more importantly, we emphasize a community-dwelling population, which contrasts with the studies focusing on individuals with specific diseases. This distinction underscores the broader impact of nutritional status on dynapenia, potentially independent of underlying medical conditions. Several possible explanations exist for these findings. First, malnutrition in older adults can result from inadequate dietary protein intake [28,36] due to age-associated alterations in sensory function and metabolism [28,48], which influence muscle protein synthesis pathways and impair muscle growth and repair [49], ultimately declining muscle strength and functional capacity in aging populations [22,50,51]. Second, older adults at malnutrition risk may be more likely to experience vitamin D deficiency [49,52], which plays a critical role in muscle function by promoting calcium absorption and facilitating protein synthesis [53]. Vitamin D deficiency often presents with marked proximal muscle weakness [54], and some studies have accordingly established an association between this deficiency and reduced muscle strength [55,56]. Third, the risk of malnutrition could exacerbate chronic inflammation in older adults [25,36], with elevated inflammatory markers potentially promoting muscle breakdown, inhibiting muscle synthesis, and further decreasing muscle strength in older adults [57,58,59]. Given that poor nutritional status may elevate the risk of dynapenia through age-related physiological factors, future research is suggested to understand these mechanisms and develop targeted interventions to prevent and manage dynapenia in aging populations.

Age moderates the relationship between nutritional status and dynapenia, with a significant association observed in the old–old (≥75 years), but not in the young–old (65–74 years) population. These findings are similar to previous studies [39,60,61,62]. A study conducted in Korea found that old–old individuals exhibited poorer gait function compared to young–old individuals [39]. Similarly, a study from Japan demonstrated that declines in handgrip strength and walking speed became more pronounced after the age of 70 [62]. Additionally, a cross-sectional study on hospitalized older adults revealed that individuals aged 75 and older had significantly poorer nutritional status than those younger than 75 [61]. Furthermore, a study from New Zealand reported that the odds of nutritional risk increased with each additional year of age and identified a significant association between lower nutritional risk and better physical performance [60]. These findings indicate that old–old individuals are disproportionately affected by the combined effects of malnutrition and muscle function decline compared to young–old individuals. Considering that aging is a complex process that affects physical, mental, and social health dimensions [28,63], there are likely underlying reasons for this relationship. First, from a physiological perspective, aging accelerates muscle fiber atrophy and loss, significantly reducing muscle mass and strength [37] with decreases in muscle strength often preceding losses in muscle mass [20,64]. As the age advances, reduced gastrointestinal function and diminished appetite often occur due to sensory changes, potentially leading to inadequate nutrient intake and a heightened risk of malnutrition [23,25]. In addition, aging correlates with an elevated risk of chronic diseases and persistent low-grade inflammation, both of which pose challenges to nutrient absorption [36] and heighten the risk of dynapenia [10]. From a psychosocial perspective, aging presents emotional and social challenges, including increased risks of depression, anxiety [24,65], and reduced social participation [66]. These factors collectively contribute to a decline in appetite [28,67] as well as in physical activity levels [68,69]. Therefore, early identification and intervention for malnutrition risk, especially in those aged over 75, may help prevent dynapenia.

This study applied the latest 2019 updated AWGS criteria to classify dynapenia, specifically tailored to older Asian populations. This approach enhanced the study’s validity by ensuring that the classification standards aligned with region-specific health characteristics, thereby improving the applicability of the findings. Additionally, the use of these criteria provides a foundation for international comparisons, enabling future research to examine dynapenia prevalence and characteristics across different Asian countries. Certain limitations were present in this study. First, the recruitment of participants was conducted in a highly urbanized region in Taiwan, limiting the generalizability of our findings to the entire Taiwanese population. Second, the cross-sectional methodology restricted the ability to infer causal relationships. Third, the MNA-SF depends primarily on self-reported data and lacks objective measurements of food intake, which may affect the accuracy of the results.

## 5. Conclusions

Age is a potential moderator of the relationship between nutritional status and dynapenia in Taiwanese older adults living in community settings. Old–old individuals (over 75 years) who were at nutritional risk had about four times the likelihood of being at risk of dynapenia, whereas this relationship was not observed in young–old individuals (65 to 74 years). Given that the aging population is expanding globally, addressing the intersection of nutritional status and dynapenia has become a pressing public health priority. Our findings offer insights for monitoring nutritional status and implementing targeted interventions to prevent dynapenia in older adults, particularly those over 75 years old. Future studies should explore the underlying physiological mechanisms linking malnutrition to muscle strength decline and evaluate the effectiveness of targeted nutritional strategies.

## Figures and Tables

**Table 1:** Descriptive statistics of categorical and continuous variables for all participants.

Variables	Total (n = 211)	Non-Dynapenia (n = 88)	Dynapenia (n = 123)	*p*-Value
Categorical Variables ^a^	**N (%)**	**N (%)**	**N (%)**	
Age group (years)				
65–74	53 (25.1)	22 (25.0)	31 (25.2)	0.973
75+	158 (74.9)	66 (75.0)	92 (74.8)	
Sex				
Men	97 (46.0)	44 (50.0)	53 (43.1)	0.321
Women	114 (54.0)	44 (50.0)	70 (56.9)	
Educational level				
Lower than university	108 (51.2)	31 (35.2)	77 (62.6)	<0.001 *
University and over	103 (48.8)	57 (64.8)	46 (37.4)	
Living status				
Alone	20 (9.5)	8 (9.1)	12 (9.8)	0.871
With others	191 (90.5)	80 (90.9)	111 (90.2)	
Smoking				
No	194 (91.9)	85 (96.6)	109 (88.6)	0.036 *
Yes	17 (8.1)	3 (3.4)	14 (11.4)	
Alcohol drinking				
No	189 (89.6)	83 (94.3)	106 (86.2)	0.056
Yes	22 (10.4)	5 (5.7)	17 (13.8)	
MVPA (mins/per week)				
<150	145 (68.7)	50 (56.8)	95 (77.2)	0.002 *
≥150	66 (31.3)	38 (43.2)	28 (22.8)	
Nutritional status				
Normal	168 (79.6)	77 (87.5)	91 (74.0)	0.016 *
At risk	43 (20.4)	11 (12.5)	32 (26.0)	
Continuous variables ^b^	Mean (SD)	Mean (SD)	Mean (SD)	*p*-value
Age (years)	80.69 (7.06)	80.25 (7.16)	81.00 (7.00)	0.448
BMI (kg/m^2^)	24.39 (3.67)	23.64 (3.15)	24.93 (3.92)	0.009 *
MNA-SF (scores)	12.62 (1.68)	12.90 (1.43)	12.42 (1.81)	0.035 *
MVPA (mins/per week)	104.17 (167.85)	173.27 (219.32)	54.74 (91.04)	<0.001 *

^a^ Chi-square test for associations with dynapenia; * *p* < 0.05, significant. ^b^ T-test for associations with dynapenia; *p* < 0.05, significant. Abbreviations: SD, standard deviation; BMI, body mass index; MNA-SF, Mini-Nutritional Assessment Short Form; MVPA, moderate-to-vigorous physical activity.

**Table 2:** A binary logistic regression analysis of the relationship between MNA-SF, nutritional status, and dynapenia in the total sample (n = 211).

Variables	Model 1		Model 2	
	OR (95%CI)	*p*-Value	OR (95%CI)	*p*-Value
Nutritional status at risk (Ref. Normal)	2.46 (1.16, 5.21)	0.018 *	3.11 (1.31, 7.36)	0.009 **
MNA-SF (scores)	0.83 (0.70, 1.00)	0.045 *	0.73 (0.57, 0.93)	0.011 *

Model 1: unadjusted; model 2: adjusted for age, sex, educational level, BMI, smoking status, and MVPA. Abbreviations: Ref., reference; OR, odds ratio; CI, confidence interval. * *p* < 0.05. ** *p* < 0.01.

**Table 3:** Significance of interactions between nutritional status and characteristic variables by binary logistic regression model.

Variables	*p*-Value for Interaction Termwith Nutritional Status
	Dynapenia (*p*-Value)
Age	0.014 *
Sex	0.125
Educational level	0.863
Living status	0.675
Smoking	0.999
Alcohol drinking	0.999

* *p* < 0.05.

**Table 4:** Age-stratified binary logistic regression models of nutritional status and dynapenia.

Variables	Model 1		Model 2	
	OR (95%CI)	*p*-Value	OR (95%CI)	*p*-Value
Aged 65–74 (n = 53)				
Nutritional status at risk (Ref. Normal)	1.31 (0.33, 5.18)	0.698	1.81 (0.35, 9.40)	0.480
Aged 75+ (n = 158)				
Nutritional status at risk (Ref. Normal)	3.15 (1.27, 7.80)	0.013 *	4.11 (1.39, 12.15)	0.010 *

Model 1: unadjusted; model 2: adjusted for sex, educational level, BMI, smoking status, and MVPA. Abbreviations: Ref., reference; OR, odds ratio; CI, confidence interval. * *p* < 0.05.

## Data Availability

The original contributions presented in the study are included in the article, further inquiries can be directed to the corresponding author.
